# Monocytes From Patients With Macrophage Activation Syndrome and Secondary Hemophagocytic Lymphohistiocytosis Are Hyperresponsive to Interferon Gamma

**DOI:** 10.3389/fimmu.2021.663329

**Published:** 2021-03-17

**Authors:** Antonia Pascarella, Claudia Bracaglia, Ivan Caiello, Alessia Arduini, Gian Marco Moneta, Marianna Nicoletta Rossi, Valentina Matteo, Manuela Pardeo, Fabrizio De Benedetti, Giusi Prencipe

**Affiliations:** ^1^ Division of Rheumatology, IRCCS Ospedale Pediatrico Bambino Gesù, Rome, Italy; ^2^ Laboratory of Immuno-Rheumatology, IRCCS Ospedale Pediatrico Bambino Gesù, Rome, Italy

**Keywords:** hemophagocytic lymphohistiocytosis, IFNγ, STAT1, macrophage activation syndrome, monocytes

## Abstract

**Objective:**

To investigate the activation of the IFNγ signaling pathway in monocytes of patients with secondary hemophagocytic lymphohistiocytosis (sHLH)/macrophage activation syndrome (MAS) and to evaluate whether levels of phosphorylated STAT1 represent a biomarker for the identification of patients at early stages of the disease.

**Methods:**

Fresh whole blood samples from pediatric patients with active sHLH/MAS, not receiving (n=10) and receiving glucocorticoids (n=14) at time of sampling, were prospectively collected. As disease control groups, patients with active systemic juvenile idiopathic arthritis (sJIA) without MAS, patients with sHLH/MAS in remission and patients with other rheumatic diseases were also sampled. Whole blood cells were left unstimulated or stimulated with increasing concentrations of IFNγ for 10 minutes and the intracellular Tyrosine (701)-phosphorylated STAT1 (pSTAT1) levels were evaluated in monocytes by flow cytometry.

**Results:**

Monocytes from untreated sHLH/MAS patients showed significantly higher basal levels of pSTAT1 compared to those observed in monocytes from glucocorticoid-treated sHLH/MAS patients and from all the other disease controls. In addition, a significant increase in responsiveness to IFNγ, as assessed by increased levels of pSTAT1 following *ex vivo* stimulation, was observed in monocytes from untreated sHLH/MAS patients. pSTAT1 levels in monocytes distinguished patients with sHLH/MAS not treated with glucocorticoids from patients with active sJIA or with other rheumatic diseases [AUC, 0.93; 95% confidence interval 0.85-1.00, p<0.001]. Statistically significant correlations between *IFNG* mRNA levels in whole blood cells, circulating IFNγ levels and pSTAT1 levels in sHLH/MAS monocytes were found.

**Conclusion:**

Our data demonstrating higher basal levels of pSTAT1 as well as a hyperreactivity to IFNγ stimulation in monocytes from patients with sHLH/MAS point to perturbations in the activation of downstream IFNγ signaling pathway as a contributor to the hyperinflammation occurring in these patients. Finally, the observation that glucocorticoids affect pSTAT1 levels *in vivo*, makes it difficult to consider the measurement of pSTAT1 levels as a biomarker to identify patients at early stages of sHLH/MAS in clinical practice.

## Introduction

Hemophagocytic lymphohistiocytosis (HLH) is a life-threatening syndrome characterized by hyperinflammation caused by uncontrolled activation of immune cells, particularly monocytes/macrophages and CD8+ T cells. It is classified into primary and secondary forms, depending on whether they are inherited or acquired (1). Indeed, while primary HLH (pHLH) is caused by loss-of-function mutations in genes encoding proteins involved in the cytolytic secretory functions of natural killer (NK) cells and cytotoxic T lymphocytes, secondary HLH forms (sHLH)/macrophage activation syndrome (MAS) often occur as complication of malignancies, infections and rheumatic diseases (2).

A growing body of evidence demonstrated the involvement of IFNγ in the pathogenesis of primary and secondary HLH as a major regulator of macrophage hyperactivation and hemophagocytosis (3–6). Further pointing to the role of IFNγ in the development of sHLH, elevated levels of IFNγ and IFNγ-regulated chemokines CXCL9 and CXCL10 have been found in the blood and in target tissues of patients and animal models (5, 7–10). Therapeutic neutralization of IFNγ has been shown to be efficacious in the treatment of primary and secondary HLH forms (11–13).

IFNγ is a pleiotropic cytokine with a key role in the regulation of innate and adaptive immune responses. It is mainly produced by activated NK and T cells and is involved in the activation of macrophages and NK cells, as well as in the differentiation and proliferation of T cells (14). IFNγ exerts its biological effects by activation of the Janus kinase (JAK)/signal transducer and activator of transcription (STAT) signal transduction pathway (15). Upon binding of IFNγ to its receptor, JAK1 and JAK2 are recruited and phosphorylated at the intracellular domain of the receptor. This event allows the recruitment of STAT1 and its activation *via* the JAKs-mediated phosphorylation of the tyrosine 701 residue (Y701). Finally, activated STAT1 translocates into the nucleus and initiates the transcription of IFNγ inducible genes (16).

In this study, we evaluated the activation of the IFNγ signaling pathway in monocytes of patients with sHLH/MAS, by assessing the intracellular levels of STAT1-Y701 phosphorylation (pSTAT1), under both basal conditions and following *ex vivo* stimulation with IFNγ. We aimed to investigate the activation of the IFNγ signaling pathway in monocytes of patients with sHLH/MAS and to evaluate whether levels of pSTAT1 could be used in a clinical setting to identify patients at early stages of HLH/MAS.

## Methods and Patients

### Patients

Patients were classified as follows. sJIA patients were diagnosed according to the ILAR criteria (17, 18). The 2016 classification criteria for MAS in sJIA were used to define patients with MAS in the context of sJIA (19). The 2004-HLH diagnostic guidelines were used to define those with sHLH (20). Patients who experienced an episode of MAS or sHLH, but were in clinical and laboratory remission at the time of whole blood collection, were also enrolled in the study. Since there are no criteria available to define disease remission of sHLH and MAS, we used those applied in the context of the clinical trial with the anti- IFNγ monoclonal antibody (emapalumab) in primary HLH and in MAS in the context of sJIA, respectively (11, 21).

The study was reviewed and approved by the local Institutional Ethical Committee of Bambino Gesù Children’s Hospital (number 1649/2018). Written informed consent to participate in this study was provided by the participants’ legal guardian/next of kin.

### Flow Cytometry

Fresh peripheral whole blood cells were left unstimulated or stimulated with different concentrations of human recombinant IFNγ (0.01, 0.1, 1, 10 ng/ml) (R&D Systems) for 10 minutes at 37°C. Anti-CD3, anti-CD14 and anti-CD16 (all from Becton Dickinson) staining was performed for 20 minutes at 4°C, in order to discriminate the monocyte, neutrophil, natural killer and T cell subpopulations. Whole blood cells where then fixed with Lyse/Fix Buffer 10 min at 37°C and further incubated 10 min at RT with FcBlock 1:200 in Stain Buffer (all from Becton Dickinson). After permeabilization with Perm Buffer II (BD PhosFlow) 20 min at 4°C, samples were stained with antibodies against phosphorylated Tyrosine (701) STAT1 (pSTAT1) and total STAT1 (all from Becton Dickinson) for 20 min at 4°C. Isotype-matched control mAbs were used to determine non-specific background staining. Samples were run on a BD LSRFortessa X‐20 instrument (BD Biosciences) and data were analyzed with FlowJo software, version 8.3 (Tree Star). Results were expressed as Delta mean fluorescence intensity (ΔMFI, calculated by subtracting MFI values of isotype controls from sample MFI values).

### Real-Time PCR

Whole blood samples were collected in Tempus Tube (Applied Biosystems). RNA was extracted with Tempus Spin RNA isolation kit (Applied Biosystems). Total RNA was reverse-transcribed with Superscript VILO cDNA synthesis Kit (Invitrogen). Gene expression levels were evaluated by quantitative polymerase chain reaction (qPCR) (ABI Prism 7900 HT sequence detection platform, Applied Biosystems), with Taqman Universal PCR Mastermix and Gene-expression Assays (Applied Biosystems; *IFNG*, Hs00989291_m1). The results, determined using the 2^−ΔCt^ method, were normalized using GAPDH (Hs99999905_m1; Applied Biosystems) as endogenous control.

### IFNγ Measurements

Plasma levels of IFNγ were measured using the IFNγ quantikine Elisa Kit (R&D Systems Inc.). The detection limit of the assays was 15.6 pg/ml.

### Statistical Analysis

Normality was tested using the Kolmogorov-Smirnov test. For normally distributed data, differences between groups were analyzed by one-way or two-way analysis of variance (ANOVA) test with Bonferroni’s correction. Data are expressed as mean ± standard error of the mean (SEM). For non-normally distributed data, differences between groups were analyzed by the nonparametric Mann–Whitney U test and data are expressed as median and interquartile range (IQR). Correlations were tested using Spearman’s rank order test. Significance level for all statistical tests was at *p < 0.05, **p < 0.01, ***p < 0.001 and ****p < 0.0001 values. Graphpad Prism 9 software was used for statistical analysis and graphs.

## Results

### Patients

Sixty-one patients were enrolled from 2017 to 2019 at Ospedale Pediatrico Bambino Gesù: patients with active sJIA (n=12; age 10.3 years, IQR 5.7-14.2; 4/12 treated with glucocorticoids), patients with sHLH/MAS not treated with glucocorticoids (n=10; age 7.1 years, IQR 5.1-14.04/12; 0/10 treated with glucocorticoids), patients with sHLH/MAS treated with glucocorticoids (n=14; age 6.3 years, IQR 2.5-14.9; 14/14 treated with glucocorticoids), patients with sHLH/MAS in remission phase (n=12; age 11.8 years, IQR 5.7-17.8; 2/12 treated with glucocorticoids), patients with other rheumatic diseases (n=13; age 6.2 years, IQR 4.3-12.7; 0/13 treated with glucocorticoids). Between 26 patients with sHLH/MAS enrolled, 13 patients experienced MAS in sJIA and 13 patients experienced sHLH, of which 5 secondary to infections, 4 secondary to inflammatory disorders, 2 secondary to malignancy, 2 of unknown origin. Between 13 patients with other rheumatic diseases enrolled, 2 patients with polyarticular juvenile idiopathic arthritis (JIA) antinuclear antibodies (ANA) positive, 5 patients with oligoarticular JIA (3 of whom with ANA positive and 2 with ANA negative), 1 with chronic non-bacterial osteomyelitis, 3 with undefined autoinflammatory diseases, 1 with undefined orbital granulomatous lesions and 1 with Kikuchi-Fujimoto disease. Patients’ laboratory parameters are shown in **Table 1**.

**Table 1:** Patient laboratory parameters.

	Other inflam.diseases (n=13)	Active sJIA patients (n=12)	Untreated sHLH/MAS (n=10)	Treated sHLH/MAS (n=14)	Remission sHLH/MAS (n=12)
**White blood cells (cells/mL)**	6500 (5815-7950)	9980 (6175-14408)	3880 (2060-6323)	6935 (2933-20798)	6145 (4093-8018)
**Hemoglobin (gr/dL)**	12.7 (11.65-13.2)	11 (9.6-11.3)	9.9 (9.2-11)	9.9 (8.4-10.6)	11.9 (10.2-12.8)
**Platelets (x10^9/L)**	277 (260-349.5)	284 (232-370)	124 (63.7-225.5)	133.5 (52.5-442.5)	244 (152.3-369.3)
**Ferritin (ng/mL)**	NA	327 (149-1457)	3718 (1270-10535)	2612 (1212-17682)	23.5 (15.7-136.4)
**D-dimer (mg/mL)**	NA	1.94 (1.2-3.4)	3.1 (2.0-6.1)	2.2 (0.82-6.1)	0.4 (0.3-1.2)
**Fibrinogen (mg/dL)**	NA	444.5 (361-626)	379.5 (191.8-493.3)	356 (228.3-552.3)	264 (232-381)
**ALT (U/L)**	23.5 (15.5-31.8)	15 (10.2-22)	59 (23-118.8)	129 (46-689.3)	27.5 (16.5-57.5)
**AST (U/L)**	29 (22.5-31.5)	32.5 (18.2-49.7)	80.5 (49.7-137.8)	67 (43.2-345.5)	31.5 (20.5-79.7)
**LDH (U/L)**	452.5 (396.5-522.8)	545 (345-684)	1066 (688.5-1848)	886.5 (609.8-1015)	499.5 (379.5-700.3)
**GammaGT (U/L)**	10 (7-19.2)	12.5 (9.25-19.3)	34.5 (14.2-123)	127.5 (53.5-313)	16 (9.2-52.2)
**CRP (mg/dL)**	0.57 (0.1-1.3)	4.7 (1.4-11.7)	6.9 (1.5-16.8)	3.2 (1.2-17.34)	0.2 (0.05-2.4)

Values are shown as median and interquartile range (IQR). NA, not available.

### Increased Levels of Phosphorylated STAT1 in Monocytes From Untreated sHLH/MAS Patients

**Figure 1:**
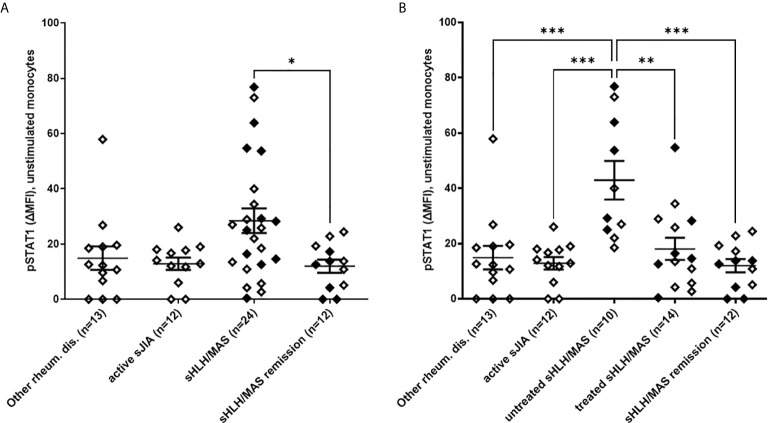
Increased phosphorylated STAT1 (pSTAT1) levels in monocytes of untreated sHLH/MAS patients. **(A)** pSTAT1 levels were evaluated in unstimulated monocytes from sHLH/MAS patients and compared to levels observed in monocytes from patients with other rheumatic diseases, patients with active sJIA, patients with sHLH/MAS in a remission phase. **(B)** pSTAT1 levels were evaluated in sHLH/MAS patients not treated (untreated sHLH/MAS) and treated (treated sHLH/MAS) with glucocorticoids at sampling and compared with the other patient groups. Full diamonds indicate samples from MAS patients. **(A, B)** pSTAT1 levels were measured by flow cytometry and reported as Delta mean fluorescence intensity (ΔMFI, calculated by subtracting MFI values of isotype controls from sample MFI values). Differences between groups were analyzed using the one-way ANOVA test and the *post hoc* Bonferroni’s for multiple comparisons test. The data shown represent mean ( ± SEM) values. *p ≤ 0.05, **p ≤ 0.01, ***p ≤ 0.001.

To evaluate the *in vivo* activation of the IFNγ pathway in patients with sHLH/MAS and to investigate whether levels of activated STAT1 represent a biomarker for the identification of patients at early stages of the disease, we measured basal activated Tyrosine (701) phosphorylated STAT1 (pSTAT1) levels in monocytes of patients with sHLH/MAS at time of blood sampling. These levels were compared with those of patients with active sJIA without MAS, with sHLH/MAS in remission phase, and with other inflammatory rheumatic diseases. sHLH/MAS patients showed significantly increased pSTAT1 levels compared to sHLH/MAS patients in remission and a trend to higher levels of pSTAT1 compared to active sJIA patients (**Figure 1A**). This was true for both sHLH and MAS patients.

Based on several data demonstrating that, *in vivo* and *ex vivo*, glucocorticoids inhibits IFNγ-mediated phosphorylation of STAT1 (9, 22), we divided sHLH/MAS patients into two groups: patients without any treatment at time of blood sampling (untreated patients, n=10) and patients who were already receiving glucocorticoids (treated patients, n=14). We found that untreated sHLH/MAS patients showed significantly higher basal pSTAT1 levels in monocytes compared to treated sHLH/MAS patients, as well as to all the other disease control groups (**Figure 1B**).

### Monocytes From Untreated MAS/sHLH Patients Show Increased Responsiveness to IFNγ

**Figure 2:**
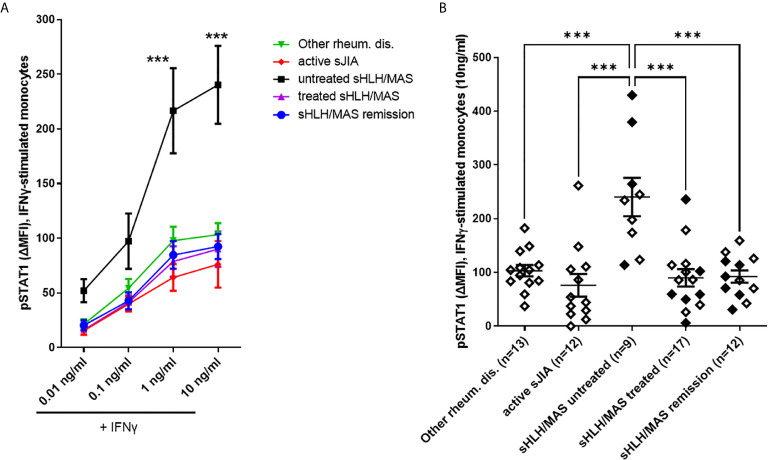
Monocytes from untreated MAS/sHLH patients show increased responsiveness to IFNγ. **(A)** pSTAT1 levels were evaluated in monocytes from patients following *ex vivo* stimulation with increasing concentration of human recombinant IFNγ. Differences between groups were analyzed using the two-way ANOVA test and the *post hoc* Bonferroni’s for multiple comparisons test. The data shown represent mean ( ± SEM) values; ***p ≤ 0.001. In untreated sHLH/MAS patients, pSTAT1 levels following stimulation with 1 and 10 ng/ml of IFNγ were significantly higher than those observed in all the other patient groups (p < 0.0001). **(B)** pSTAT1 levels in monocytes stimulated with 10 ng/ml of IFNγ are reported in detail. Full diamonds indicate samples from MAS patients. **(A, B)** pSTAT1 levels were measured by flow cytometry and reported as Delta mean fluorescence intensity (ΔMFI, calculated by subtracting MFI values of isotype controls from sample MFI values). Untreated sHLH/MAS patients were 9 instead of 10, because for one patient there were not enough cells for IFNγ-stimulation experiments.

To investigate potential perturbations in IFNγ signaling pathway activation in sHLH/MAS, we analyzed pSTAT1 levels in monocytes following *ex vivo* stimulation with increasing concentrations of IFNγ. As shown in **Figure 2**, we found that monocytes of untreated sHLH/MAS patients showed a significant increase in responsiveness to IFNγ stimulation (at 1 and 10 ng/ml of IFNγ), compared to all the other disease control groups. Notably, following IFNγ stimulation, monocytes from glucocorticoid-treated sHLH/MAS patients showed pSTAT1 levels superimposable to those of the other patient/control groups. In monocytes of untreated sHLH/MAS patients, levels of pSTAT1 following IFNγ stimulation were significantly correlated with basal pSTAT1 levels (IFNγ 10 ng/ml: p=0.01, r=0.77). This correlation was not observed in disease control groups. We also found a correlation of pSTAT1 levels in unstimulated (n=10, p=0.01, r=0,75) and IFNγ (10 ng/ml) stimulated monocytes (n=10, p=0.03, r=0.68) with *IFNG* mRNA expression in whole blood paired samples. Consistently, plasma levels of IFNγ were high in patients with sHLH/MAS (mean± SEM, 51 pg/ml ± 14.7, n=24) and were correlated with pSTAT1 levels in monocytes not stimulated (n=24, p=0.004, r=0.56) or stimulated with IFNγ (n=23, p<0.0001, r=0.72). These data suggest a relation between overexpression of IFNγ *in vivo* and enhanced pSTAT1 levels *ex vivo*. In contrast, in patients with active sJIA, circulating levels of IFNγ were undetectable and no statistically significant correlations between IFNG mRNA, circulating levels of IFNγ and pSTAT1 levels were observed.

**Figure 3:**
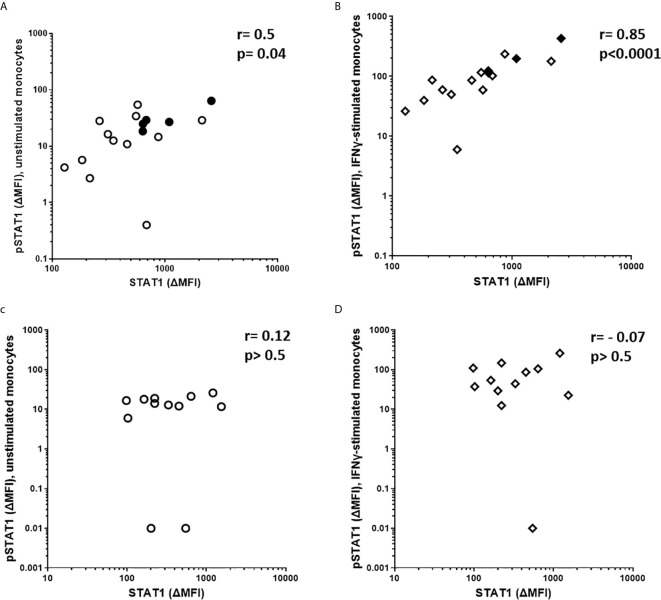
Total STAT1 levels are significantly correlated with pSTAT1 levels in monocytes of sHLH/MAS patients, but not in those of active sJIA patients. **(A, B)** Total STAT1 levels were correlated with pSTAT1 levels in monocytes unstimulated (n=17) or stimulated (n=16) with IFNγ (10 ng/Mml) from sHLH/MAS patients. Full diamonds and circles indicate samples from untreated sHLH/MAS patients. **(C, D)** Total STAT1 levels were correlated with pSTAT1 levels in monocytes unstimulated (n=12) or stimulated (n=12) with IFNγ (10ng/ml) from active sJIA patients. Correlations were tested using Spearman’s rank order test. A base-10 log scale is used for the y and x axes of graphs **(A–D)**.

We also measured by flow cytometry total STAT1 expression in unstimulated monocytes from a smaller number of patients. Due to the small number of samples analyzed, we found a trend for higher levels of STAT1 in untreated sHLH/MAS patients (**Supplementary Figure 1**). However, we found that total STAT1 levels significantly correlated with pSTAT1 levels in both unstimulated (p=0.04, r=0.5) (**Figure 3A**) and IFNγ-stimulated sHLH/MAS monocytes (p<0.0001, r=0.85) (**Figure 3B**). In contrast, no significant correlations were observed between total STAT1 levels and pSTAT1 levels in patients with active sJIA (**Figures 3C, D**).

Altogether, these results showed significantly higher levels of pSTAT1 both in unstimulated and *ex vivo* IFNγ-stimulated monocytes from patients with sHLH/MAS who were not receiving glucocorticoids. These levels correlated with mRNA expression and circulating levels of IFNγ. In addition, a correlation between total STAT1 levels and pSTAT1 levels in unstimulated and IFNγ–stimulated monocytes from sHLH/MAS not receiving glucocorticoids was also found. These results are consistent with the upregulation of IFNγ and the activation of IFNγ-mediated signaling pathway in patients with sHLH/MAS.

### Clinical Relevance of pSTAT1 Levels in sHLH/MAS

**Figure 4:**
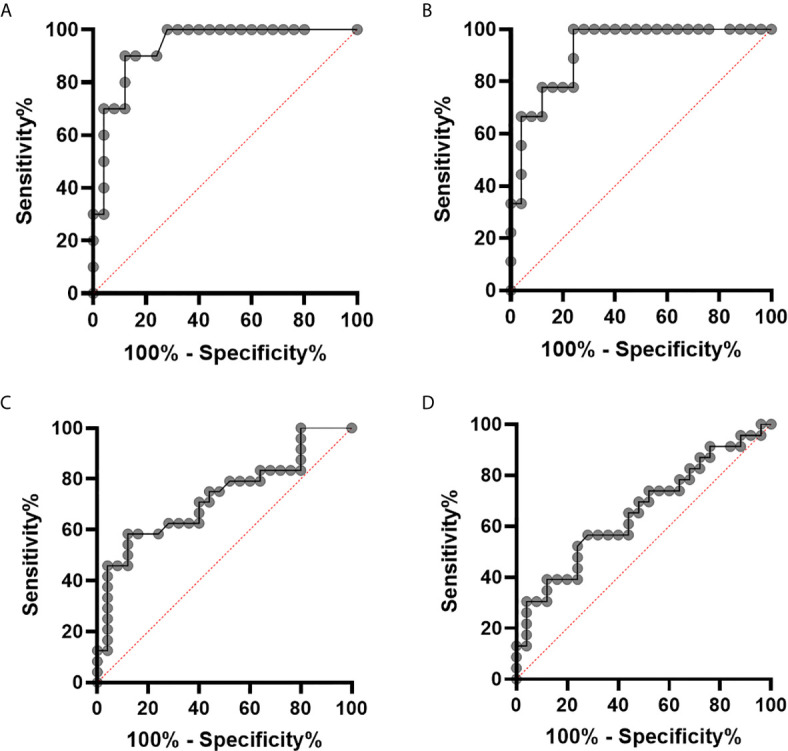
pSTAT1 levels in monocytes distinguish patients with sHLH/MAS not treated with glucocorticoids at sampling from patients with active sJIA or with other rheumatic diseases. **(A, B)** receiver operating characteristic (ROC) curve of pSTAT1 levels in unstimulated **(A)** and IFNγ (10 ng/ml)–stimulated **(B)** monocytes distinguishing patients with sHLH/MAS not treated with glucocorticoids at sampling from patients with active sJIA or with other rheumatic diseases. **(C, D)** ROC curve of pSTAT1 levels in unstimulated **(C)** and IFNγ (10 ng/ml)–stimulated **(D)** monocytes distinguishing all patients with sHLH/MAS (not treated and treated with glucocorticoids at sampling) from patients with active sJIA or with other rheumatic diseases.

In order to evaluate whether pSTAT1 levels could be used to identify HLH/MAS patients in a clinical setting, we performed receiver operating characteristic (ROC) curve analysis, by using as controls patients with active sJIA without MAS and patients with other rheumatic diseases. When untreated sHLH/MAS patients were analyzed, we found an area under the curve (AUC) of 0.93 [95% confidence interval (CI), 0.85-1.00, p<0.001] for pSTAT1 levels in unstimulated monocytes (**Figure 4A**) and an AUC of 0.92 (95% CI, 0.83-1.00, p<0.001) for pSTAT1 levels in monocytes stimulated with IFNγ (10 ng/ml) (**Figure 4B**). However, when all sHLH/MAS patients (untreated and treated with glucocorticoids at sampling) were analyzed, we found an AUC of 0.73 (95% CI, 0.58-0.87, p=0.006) for pSTAT1 levels in unstimulated monocytes (**Figure 4C**) and an AUC of 0.66 (95% CI, 0.50-0.81, p=0.06) for pSTAT1 levels in monocytes stimulated with IFNγ (10 ng/ml) (**Figure 4D**).

When we evaluated in untreated MAS/sHLH patients and in all MAS/sHLH patients (treated and untreated) the correlations of pSTAT1 and total STAT1 with clinical and laboratory parameters, including white blood cell count, platelet count, hemoglobin, ferritin, fibrinogen, we did not find any statistically significant correlation (data not shown).

## Discussion

A growing number of studies point to a pivotal role of IFNγ in the pathogenesis of primary and secondary HLH forms, including MAS (4, 7).

Consistently with the observations of high levels of IFNγ and IFNγ-inducible inflammatory mediators in blood of patients and in animal models with sHLH (5–7), we found that freshly isolated blood monocytes from patients with sHLH/MAS show increased levels of pSTAT1, further confirming the activation of the IFNγ signaling pathway in these patients. In particular, we found that blood monocytes of patients with sHLH/MAS not treated with glucocorticoids express significantly higher levels of pSTAT1 compared to patients with active sJIA, as well as compared to patients with sHLH/MAS treated with glucocorticoids or to sHLH/MAS patients during remission. In addition, we found a statistically significant correlation of total STAT1 levels with pSTAT1 levels in sHLH/MAS patients. These data are consistent with previous findings demonstrating that STAT1 and pSTAT1 levels are strongly increased in target tissues, such as liver and lung, of patients and animals with sHLH/MAS (5, 8, 9).

Our data showing significant differences in pSTAT1 levels between untreated patients and glucocorticoid-treated patients are consistent with the known inhibitory effects, both *in vitro* and *in vivo*, of glucocorticoids on the IFNγ-mediated signaling pathway. Indeed, addition of dexamethasone to PBMC cultures has been demonstrated to result in the suppression of STAT1 expression and, therefore, in a dramatic inhibition of IFNγ signaling pathway activation, as revealed by suppression of IFNγ-inducible gene expression (22). Our observation also confirms our previous findings in PBMCs collected from one patient with sHLH: total STAT1 and pSTAT1 levels were significantly increased during the acute phase of the disease, but they underwent a marked decrease following initiation of treatment with glucocorticoids (9). Glucocorticoids are often used in the early phase of the disease to stabilize patients with rapidly progressive worsening disease. Indeed, we found that pSTAT1 levels in unstimulated and IFNγ-stimulated monocytes are sensitive and specific in discriminating sHLH/MAS patients not treated with glucocorticoids at sampling from patients with active sJIA and other rheumatic diseases. In contrast, they do not distinguish sHLH/MAS patients treated with glucocorticoids at sampling from disease controls. Altogether, these observations make it difficult to consider the measurement of pSTAT1 levels as a good biomarker for the identification of patients at early stages of sHLH/MAS in clinical practice.

To investigate potential perturbations in downstream IFNγ signaling pathway activation in monocytes of patients with sHLH/MAS, we also evaluated the activation of STAT1 following *ex vivo* stimulation of peripheral whole blood cells with increasing concentrations of recombinant IFNγ. We found that IFNγ stimulation was much more effective in inducing STAT1 phosphorylation in monocytes of sHLH/MAS patients not treated with glucocorticoids compared to all the other patient groups analyzed. These results are consistent with *in vitro* findings, demonstrating that in macrophages exposition to subthreshold concentrations of IFNγ increased their sensitivity to subsequent IFNγ stimulation, resulting in increased pSTAT1 and increased IFNγ–dependent gene expression (23). It is therefore conceivable that in patients with sHLH/MAS *in vivo* pre-exposure of monocytes to IFNγ (7) led to hyper-response to subsequent *ex vivo* IFNγ stimulation. Accordingly, we observed a correlation between *IFNG* mRNA expression levels in whole blood cells, circulating IFNγ levels and pSTAT1 levels in unstimulated and IFNγ-stimulated monocytes of sHLH/MAS patients. The exact mechanism involved in the hyper-responsiveness to IFNγ of monocytes from sHLH/MAS patients remains to be elucidated. Other cytokines or a particular inflammatory milieu may affect total STAT1 levels in patients with sHLH/MAS. For example, TNF has been demonstrated to induce sustained expression of STAT1 and IFN-response genes in monocytes and macrophages (24). Recently, a role for increased TRIM8 expression in enhancing macrophage responsiveness to IFNγ in patients with active sJIA and MAS has also been reported (25).

Our results demonstrating that the activation of the IFNγ−mediated signaling pathway in patients with active sJIA without MAS and in patients with sHLH/MAS in remission is comparable to that observed in patients with other rheumatologic diseases provide evidence of a normal response of monocytes to IFNγ in these conditions. These data are in agreement with previous results obtained by Sikora et al., demonstrating that monocytes of patients with active sJIA do not show a hyperresponsiveness to IFNγ stimulation (26). In this study, we showed that, during the active phase of sHLH/MAS, monocytes are more responsive to IFNγ stimulation. These results add new insights to the pathogenesis of sHLH, demonstrating that sHLH/MAS is not only characterized by increased production of IFNγ (7), but also by increased reactivity of monocytes to IFNγ stimulation.

The major limitation of this study is the small sample size, as the study was conducted at one hospital center only. This is mainly due to the need to use freshly isolated cells for the cytofluorimetric assay, as we found that cell cryopreservation affected pSTAT1 staining.

In summary, consistently with the activation of the IFNγ pathway, we report that pSTAT1 levels are increased in monocytes of patients with HLH/MAS. Given the major effect of glucocorticoid treatment, often required to stabilize patients, on *in vivo* pSTAT1 levels and the need for freshly isolated cells and the relatively sophisticated technique, pSTAT1 levels cannot be used in routine practice as a test to identify patients at early stages of HLH/MAS. The observed hyper-response to *ex vivo* IFNγ stimulation suggests that both increased production of, and increased response to, IFNγ contribute to the amplification of hyperinflammation. Our results provide additional mechanistic support for the use of neutralizing IFNγ antibodies in the treatment of sHLH/MAS and, therefore, may have an impact on the justification of the dose of neutralizing antibody to be used.

## Data Availability Statement

The raw data supporting the conclusions of this article will be made available by the authors, without undue reservation.

## Ethics Statement

The local Institutional Ethical Committee of Bambino Gesù Children’s Hospital approved the study (number 1649/2018). Written informed consent to participate in this study was provided by the participants’ legal guardian/next of kin.

## Author Contributions

GP, CB, and FDB conceived and designed the work. AP, IC, MNR, GMM, and VM performed the experiments. CB, AA, and MP enrolled patients and collected clinical data. GP and FDB wrote the manuscript. All authors contributed to the article and approved the submitted version.

## Conflict of Interest

The authors declare that the research was conducted in the absence of any commercial or financial relationships that could be construed as a potential conflict of interest.

## Supplementary Material

Supplementary Figure 1 Total STAT1 levels in monocytes of sHLH/MAS patients. **(A)** Total STAT1 levels were evaluated in unstimulated monocytes from untreated or treated sHLH/MAS patients and compared to levels observed in monocytes from patients with other rheumatic diseases, patients with active sJIA and patients with sHLH/MAS in a remission phase. STAT1 levels were measured by flow cytometry and reported as Delta mean fluorescence intensity (ΔMFI, calculated by subtracting MFI values of isotype controls from sample MFI values). Differences between groups were analyzed using the one-way ANOVA test and the *post hoc* Bonferroni’s for multiple comparisons test. The data shown represent mean (± SEM) values.

## References

[1] Risma KA Marsh RA. Hemophagocytic Lymphohistiocytosis: Clinical Presentations and Diagnosis. J Allergy Clin Immunol Practice (2019) 7:824–32. 10.1016/j.jaip.2018.11.050 30557712

[2] Griffin G Shenoi S Hughes GC. Hemophagocytic lymphohistiocytosis: An update on pathogenesis, diagnosis, and therapy. Best Pract Res Clin Rheumatol (2020) 34:101515. 32387063 10.1016/j.berh.2020.101515

[3] Zoller EE Lykens JE Terrell CE Aliberti J Filipovich AH Henson PM. J Exp Med. Hemophagocytosis Causes Consumptive Anemia Inflam (2011) 208:1203–14. 10.1084/jem.20102538 PMC3173248 21624938

[4] Jordan MB Hildeman D Kappler J Marrack P. An animal model of hemophagocytic lymphohistiocytosis (HLH): CD8+ T cells and interferon gamma are essential for the disorder. Blood (2004) 104:735–43. 10.1182/blood-2003-10-3413 15069016

[5] Prencipe G Caiello I Pascarella A Grom AA Bracaglia C Chatel L. Neutralization of IFN-γ reverts clinical and laboratory features in a mouse model of macrophage activation syndrome. J Allergy Clin Immunol (2018) 141:1439–49. 10.1016/j.jaci.2017.07.021 28807602

[6] Behrens EM Canna SW Slade K Rao S Kreiger PA Paessler M. Repeated TLR9 stimulation results in macrophage activation syndrome-like disease in mice. J Clin Invest (2011) 121:2264–77. 10.1172/JCI43157 PMC3104738 21576823

[7] Bracaglia C de Graaf K Pires Marafon D Guilhot F Ferlin W Prencipe G. Elevated circulating levels of interferon-γ and interferon-γ-induced chemokines characterise patients with macrophage activation syndrome complicating systemic juvenile idiopathic arthritis. Ann Rheum Dis (2017) 76:166–72. 10.1136/annrheumdis-2015-209020 27296321

[8] Schulert GS Yasin S Carey B Chalk C Do T Schapiro AH. Systemic juvenile idiopathic arthritis-associated lung disease: Characterization and risk factors. Arthritis Rheumatol (2019) 71:1943–54. 10.1002/art.41073 PMC6817389 31379071

[9] Prencipe G Bracaglia C Caiello I Pascarella A Francalanci P Pardeo M. The interferon-gamma pathway is selectively up-regulated in the liver of patients with secondary hemophagocytic lymphohistiocytosis. PLoS One (2019) 14:e0226043. 31846457 10.1371/journal.pone.0226043 PMC6917341

[10] Takakura M Shimizu M Irabu H Sakumura N Inoue N Mizuta M. Comparison of serum biomarkers for the diagnosis of macrophage activation syndrome complicating systemic juvenile idiopathic arthritis. Clin Immunol (2019) 208:108252. 31449879 10.1016/j.clim.2019.108252

[11] Locatelli F Jordan MB Allen C Cesaro S Rizzari C Rao A. Emapalumab in children with primary hemophagocytic lymphohistiocytosis. N Engl J Med (2020) 382:1811–22. 10.1056/NEJMoa1911326 32374962

[12] Gloude NJ Dandoy CE Davies SM Myers KC Jordan MB Marsh RA. Thinking Beyond HLH: Clinical Features of Patients with Concurrent Presentation of Hemophagocytic Lymphohistiocytosis and Thrombotic Microangiopathy. J Clin Immunol (2020) 40:699–707. 32447592 10.1007/s10875-020-00789-4 PMC7245179

[13] Gabr JB Liu E Mian S Pillittere J Bonilla E Banki K. Successful treatment of secondary macrophage activation syndrome with emapalumab in a patient with newly diagnosed adult-onset Still’s disease: case report and review of the literature. Ann Trans Med (2020) 8:887. 10.21037/atm-20-3127 PMC7396773 32793731

[14] Schoenborn JR Wilson CB. Regulation of interferon-gamma during innate and adaptive immune responses. Adv Immunol (2007) 96:41–101. 17981204 10.1016/S0065-2776(07)96002-2

[15] Schroder K Hertzog PJ Ravasi T Hume DA. Interferon-gamma: an overview of signals, mechanisms and functions. J Leukoc Biol (2004) 75:163–89. 10.1189/jlb.0603252 14525967

[16] Saha B Jyothi Prasanna S Chandrasekar B Nandi D. Gene modulation and immunoregulatory roles of interferon gamma. Cytokine (2010) 50:1–14. 20036577 10.1016/j.cyto.2009.11.021

[17] Petty RE Southwood TR Manners P Baum J Glass DN Goldenberg J. International League of Associations for Rheumatology classification of juvenile idiopathic arthritis: second revision, Edmonton, 2001. J Rheumatol (2004) 31:390–2. 14760812

[18] Wallace CA Ruperto N Giannini E Childhood A Rheumatology Research A Pediatric Rheumatology International Trials O. Preliminary criteria for clinical remission for select categories of juvenile idiopathic arthritis. J Rheumatol (2004) 31:2290–4. 15517647

[19] Ravelli A Minoia F Davi S Horne A Bovis F Pistorio A. 2016 Classification Criteria for Macrophage Activation Syndrome Complicating Systemic Juvenile Idiopathic Arthritis: A European League Against Rheumatism/American College of Rheumatology/Paediatric Rheumatology International Trials Organisation Collaborative Initiative. Ann Rheum Dis (2016) 75:481–9. 10.1136/annrheumdis-2015-208982 26865703

[20] Henter JI Horne A Arico M Egeler RM Filipovich AH Imashuku S. HLH-2004: Diagnostic and therapeutic guidelines for hemophagocytic lymphohistiocytosis. Pediatr Blood Cancer (2007) 48:124–31. 10.1002/pbc.21039 16937360

[21] De Benedetti F BP Grom A Quartier P Schneider R Antón J Bracaglia C. Interferon-gamma (IFN-γ) Neutralization with Emapalumab and Time to Response in Patients with Macrophage Activation Syndrome (MAS) Complicating Systemic Juvenile Idiopathic Arthritis (s-JIA) who failed High-Dose Glucocorticoids abstract. Arthritis Rheumatol (2019) 71(suppl 10):5229–30.

[22] Hu X Li WP Meng C Ivashkiv LB. Inhibition of IFN-gamma signaling by glucocorticoids. J Immunol (2003) 170:4833–9. 10.4049/jimmunol.170.9.4833 12707366

[23] Hu X Herrero C Li WP Antoniv TT Falck-Pedersen E Koch AE. Sensitization of IFN-gamma Jak-STAT signaling during macrophage activation. Nat Immunol (2002) 3:859–66. 10.1038/ni828 12172544

[24] Yarilina A Park-Min KH Antoniv T Hu X Ivashkiv LB. TNF activates an IRF1-dependent autocrine loop leading to sustained expression of chemokines and STAT1-dependent type I interferon-response genes. Nat Immunol (2008) 9:378–87. 10.1038/ni1576 18345002

[25] Schulert GS Pickering AV Do T Dhakal S Fall N Schnell D. Monocyte and bone marrow macrophage transcriptional phenotypes in systemic juvenile idiopathic arthritis reveal TRIM8 as a mediator of IFN-gamma hyper-responsiveness and risk for macrophage activation syndrome. Ann Rheum Dis (2020). 10.1136/annrheumdis-2020-217470 33277241

[26] Sikora KA Fall N Thornton S Grom AA. The limited role of interferon-gamma in systemic juvenile idiopathic arthritis cannot be explained by cellular hyporesponsiveness. Arthritis Rheumatol (2012) 64:3799–808. 10.1002/art.34604 PMC3482423 22740319

