# Supplementary figures and images for: Monocytes From Patients With Macrophage Activation Syndrome and Secondary Hemophagocytic Lymphohistiocytosis Are Hyperresponsive to Interferon Gamma
Source: Front Immunol. 2021 Mar 17;12:663329. doi: 10.3389/fimmu.2021.663329 (PMC8010171; doi:10.3389/fimmu.2021.663329)

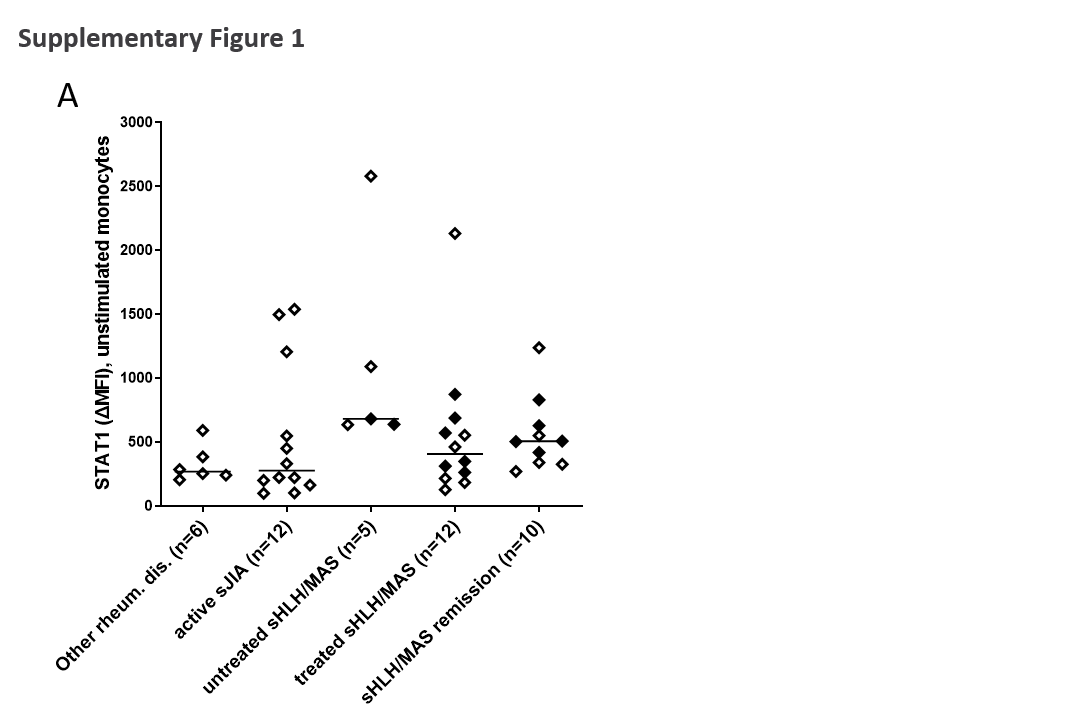

Supplement: Supplementary Figure 1 — Total STAT1 levels in monocytes of sHLH/MAS patients. (A) Total STAT1 levels were evaluated in unstimulated monocytes from untreated or treated sHLH/MAS patients and compared to levels observed in monocytes from patients with other rheumatic diseases, patients with active sJIA and patients with sHLH/MAS in a remission phase. STAT1 levels were measured by flow cytometry and reported as Delta mean fluorescence intensity (ΔMFI, calculated by subtracting MFI values of isotype controls from sample MFI values). Differences between groups were analyzed using the one-way ANOVA test and the post hoc Bonferroni’s for multiple comparisons test. The data shown represent mean (± SEM) values. [file Image_1.tif]
